# Factors that motivate individuals to volunteer to be dispatched as first responders in the event of a medical emergency: A systematic review protocol

**DOI:** 10.12688/hrbopenres.12969.2

**Published:** 2020-09-14

**Authors:** Eithne Heffernan, Iris Oving, Tomás Barry, Viet-Hai Phung, Aloysius Niroshan Siriwardena, Siobhán Masterson

**Affiliations:** 1 Discipline of General Practice, Clinical Science Institute, School of Medicine, National University of Ireland, Galway, H91 TK33, Ireland; 2 Department of Clinical and Experimental Cardiology, Amsterdam University Medical Centre, University of Amsterdam, Amsterdam, 1012 WX, The Netherlands; 3 School of Medicine, University College Dublin, Dublin 4, D04 V1W8, Ireland; 4 Community and Health Research Unit, School of Health and Social Care, Sarah Swift Building, University of Lincoln, Lincoln, LN5 7AT, UK; 5 National Ambulance Service, Health Service Executive, St. Eunan's Hall, St Conal's Hospital, Letterkenny, Donegal, F92 XK84, Ireland

**Keywords:** First response, community first responders, out-of-hospital cardiac arrest, prehospital emergency care, volunteerism, motivation, systematic review, qualitative synthesis

## Abstract

**Background: **Voluntary First Response is an important component of prehospital care for medical emergencies, particularly cardiac arrest, in many countries. This intervention entails the mobilisation of volunteers, known as Community First Responders (CFRs), by the Emergency Medical Services to respond to medical emergencies in their locality. They include lay responders and/or professional responders (e.g. police officers, fire fighters, and general practitioners). A wide variety of factors are thought to motivate CFRs to join and remain engaged in Voluntary First Response schemes, such as the availability of learning opportunities, recognition, counselling, and leadership. The aim of this review is to develop an in-depth understanding of CFR motivation, including the factors that influence the initial decision to volunteer as a CFR and the factors that sustain involvement in Voluntary First Response over time. Any factors relevant to CFR de-motivation and turnover will also be examined.

**Methods:** This is a protocol for a qualitative systematic review of the factors that influence the motivation of individuals to participate in Voluntary First Response. A systematic search will be carried out on seven electronic databases. Qualitative studies, mixed-methods studies, and any other studies producing data relating to the review question will be eligible for inclusion. Title and abstract screening, as well as full text screening, will be completed independently by two authors. A narrative synthesis, which is an established qualitative synthesis methodology, will be performed. The quality of each of the included studies will be critically appraised.

**Discussion:** The findings of this review will be used to optimise the intervention of Voluntary First Response. Specifically, the results will inform the design and organisation of Voluntary First Response schemes, including their recruitment, training, and psychological support processes. This could benefit a range of stakeholders, including CFRs, paramedics, emergency physicians, patients, and the public.

## Introduction

Volunteerism is crucial to the provision of prehospital care to patients undergoing medical emergencies, such as cardiac arrest, chest pain, and choking ( Whittaker *et al.*, 2015). It can be defined as freely and deliberately choosing to perform helping activities for causes or individuals who desire assistance in the absence of contractual or friendship/familial obligation and without expectation of financial reward ( Snyder & Omoto, 2008; Whittaker *et al.*, 2015). Volunteerism can be informal (i.e. spontaneous) or formal (i.e. organised). Informal volunteers self-deploy or offer assistance without being part of a coordinated response by a relevant authority, such as when bystanders to a cardiac arrest alert the Emergency Medical Services (EMS) and initiate cardiopulmonary resuscitation (CPR) without being part of the organised emergency response ( Geri *et al.*, 2017; Maurer *et al.*, 2019; Whittaker *et al.*, 2015). In contrast, formal volunteers provide assistance as part of their official affiliation with a relevant agency ( Whittaker *et al.*, 2015). A prime example of formal volunteers are Community First Responders (CFRs), who are dispatched by the EMS to medical emergencies in their locality ( Barry *et al.*, 2019a).

CFRs were primarily introduced to improve the management of out-of-hospital cardiac arrest (OHCA) ( Hollenberg *et al.*, 2013; Truong *et al.*, 2015). OHCA is a leading cause of mortality worldwide ( Myat *et al.*, 2018). Survival from OHCA is negligible in the absence of good-quality CPR and defibrillation within 5–10 minutes of collapse ( Monsieurs *et al.*, 2015; Ong *et al.*, 2018). Therefore, CFRs were instituted to help the EMS reduce the time taken to respond to OHCA events, particularly in rural areas ( Hollenberg *et al.*, 2013; Masterson *et al.*, 2015). These volunteers have typically completed training in basic emergency care and many carry Automated External Defibrillators ( Phung *et al.*, 2017). They can include lay responders and/or professional responders (e.g. physicians, police officers, and firefighters). A recent Cochrane review confirmed that CFR schemes can result in increased rates of CPR or defibrillation performed prior to EMS arrival ( Barry *et al.*, 2019a). Since their introduction, CFRs have come to play an integral role in OHCA management in many countries, including Canada, Japan, Australia, New Zealand, United Kingdom, and the Republic of Ireland ( Barry *et al.*, 2018; O’Meara *et al.*, 2012; Orkin *et al.*, 2016; Phung *et al.*, 2017; Toyokuni *et al.*, 2013). Furthermore, the role of CFRs has been expanded in many regions such that they are dispatched to an array of medical emergencies, including stroke, choking, and chest pain ( Phung *et al.*, 2017).

Research has uncovered a variety of factors that motivate individuals to become CFRs, including a desire to help patients, to save lives, to contribute to their local community, to contribute to health and/or social care services, to acquire or enhance knowledge and skills, to obtain experience for a future career in healthcare, to enhance their self-esteem, and to enhance their social network ( Phung *et al.*, 2017; Timmons & Vernon-Evans, 2013). In addition, some become CFRs because past experiences, such as witnessing a medical emergency or working as a healthcare professional, have given them an appreciation of the importance of first response ( Barry *et al.*, 2019b; Roberts *et al.*, 2014). There is also evidence to suggest that the motives of CFRs can be influenced by demographic factors (e.g. age, gender). For instance, an Australian study found that female volunteers tended to express altruistic values, whilst their male counterparts were more likely to express egoistic or self-oriented values ( Calcutt, 2019).

Research has provided insights on the factors that encourage individuals to maintain their participation in CFR schemes over time. For example, organising CFRs in cohesive groups that have dedicated leadership can sustain engagement ( Kasper *et al.*, 2017; Rice & Fallon, 2011). In addition, the availability of appropriate support mechanisms is crucial for the continued involvement of any CFRs who experience adverse reactions following an emergency, including stress, sleep disturbance, intrusive thoughts, and weight loss ( Kindness *et al.*, 2014; Mathiesen *et al.*, 2016; Phung *et al.*, 2018; Zijlstra *et al.*, 2015). Furthermore, it is thought that resilience can mitigate the impact of traumatic experiences on CFRs ( Timmons & Vernon-Evans, 2013). Feedback and recognition may also preserve the motivation of CFRs. Specifically, volunteers have expressed a desire for formal feedback about patient outcomes, reassurance regarding their CPR performance, and recognition of their contribution by the public and their organisation/EMS ( Mathiesen *et al.*, 2016; Phung *et al.*, 2017). Moreover, CFRs are more likely to feel valued when they have opportunities to contribute to decision-making and policy development in their organisation/EMS ( O’Meara *et al.*, 2012; Stirling & Bull, 2011).

Several studies have examined demotivation and turnover amongst CFRs. It has been reported that some CFRs find aspects of first response to be burdensome, particularly spending time away from family, being ‘on call’, and responding to night-time emergencies ( Roberts *et al.*, 2014). It can be onerous for members of small CFR groups to share responsibility for responding to emergencies with just a few volunteers ( Rørtveit & Meland, 2010). Whilst participating in first response is too time-consuming for some, other CFRs are rarely dispatched to emergencies, which can lead to deskilling and demotivation ( Timmons & Vernon-Evans, 2013). A minority of CFRs prove to be unsuitable for the role, particularly those who are motivated by the dramatic and exciting aspects of responding to emergencies and those who lack the requisite calm demeanour and interpersonal skills ( Barry *et al.*, 2019b). Finally, some CFRs have fears about performing CPR, including fears about contracting infectious diseases, causing injury, becoming embroiled in legal action, and being unable to resuscitate the patient, as well as general feelings of panic and shock ( Malta Hansen *et al.*, 2017; Savastano & Vanni, 2011).

It is vital to develop an in-depth understanding of the motivation of CFRs in order to enhance CFR recruitment and retention, to improve the training and psychological support received by CFRs, and to optimise the organisation and structure of CFR schemes. This, in turn, could improve the outcomes of patients who depend upon CFRs for their survival, as well as the significant others of those patients. One previous systematic scoping review examined the literature relating to the experiences of CFRs in the United Kingdom, including their motives for adopting the role ( Phung *et al.*, 2017). These motives included a desire to assist one’s community and to strengthen one’s employability. However, it is important to review the literature from nations other than the United Kingdom, as CFR systems vary considerably between and even within countries ( Oving *et al.*, 2019). For example, different regions utilise different categories of CFR (e.g. lay people, police officers, taxi drivers, and off-duty healthcare professionals) who are likely to have distinct motives. Furthermore, the status of CFRs varies across regions in terms of whether they are part of the EMS, complementary to the EMS, or separate to the EMS, which could also influence their motivation. Therefore, this review will expand upon its predecessor by considering the full breadth of the international literature on CFR motivation.

### Aims and objectives

This review aims to identify the factors that motivate individuals to volunteer to be dispatched as first responders in the event of a medical emergency. The specific objectives of the review are to identify: (1) the factors that influence the initial motivation of individuals to volunteer to provide first response to medical emergencies, (2) the factors that influence these individuals to sustain their voluntary participation in providing first response over time, and (3) any factors relevant to demotivation and turnover in these individuals.

## Methods

### Eligibility criteria

The inclusion criteria are specified according to the domains of the SPIDER search strategy tool: Sample, Phenomena of Interest, Design, Evaluation, and Research type ( Cooke *et al.*, 2012). The SPIDER tool was designed as an alternative to the Population, Intervention, Comparison, and Outcomes (PICO) tool, which is predominant in systematic reviews of quantitative research.

***Sample.*** The sample are Voluntary First Responders, also known as CFRs, lay rescuers, and citizen responders. They can be defined as individuals who have volunteered with the statutory ambulance services/EMS to be dispatched as a first responder in the event of a medical emergency in their locality ( Barry *et al.*, 2019a). They can include lay responders and professional responders (e.g. medical, fire service, or police personnel). They are activated by the EMS dispatch centre or equivalent (e.g. charities working with the EMS). They do not have a statutory obligation to respond to medical emergencies. Research on volunteers based in any geographical region or country will be eligible for inclusion. The review will exclude research on informal, non-dispatched Voluntary First Responders. This refers to individuals who are present at or near a medical emergency and who volunteer to provide emergency care opportunistically and spontaneously, such as when an individual who witnesses an OHCA opts to telephone the EMS and perform CPR under the instruction of an EMS call-taker.

***Phenomena of interest.*** This review will examine Voluntary First Response, which is a complex intervention for prehospital medical emergencies, including cardiac arrest, choking, stroke, and chest pain. It entails the mobilisation of individual volunteers or groups of volunteers by the EMS or equivalent as a first response to medical emergencies in their locality. Only research relating to medical emergencies in community or prehospital settings will be eligible for inclusion. Research on emergency care provided during secondary transfer of patients between hospitals will be excluded. This review will also exclude research on hospital emergencies, non-medical emergencies, military combat, man-made disasters, and natural disasters, such as fires, terrorist attacks, earthquakes, hurricanes, and nuclear disasters.

***Design.*** Primary research studies that produce data relating to the aims and objectives of the review will be considered. The methods employed in these studies can include individual interviews, group interviews, questionnaires, surveys, and observation. In addition to peer-reviewed journal articles, conference abstracts and conference proceedings relating to the review question will be considered for inclusion. The remainder of the grey literature (e.g. editorials, practice guidelines, case reports, and case series) will be excluded. Firstly, there is no agreed approach to extracting and synthesising evidence obtained from the grey literature in a transparent way. Secondly, excluding the grey literature reduces the likelihood of including poor quality studies ( Maidment *et al.*, 2018).

***Evaluation.*** This review will analyse the factors that motivate individuals to participate in Voluntary First Response for medical emergencies. This includes both the factors that influence their initial motivation to become a Voluntary First Responder and the factors that sustain their involvement in first response over time. Any factors relevant to the de-motivation (e.g. ceasing participation in first response) of Voluntary First Responders will also be reviewed. If there are sufficient data, the impact of demographic factors (e.g. age, gender) and different categories of Voluntary First Response programs (e.g. community groups, mobile application schemes, fire services, police officer schemes) will be examined.

***Research type.*** Qualitative studies, mixed-methods studies, and any other studies producing data relating to the review question will be eligible for inclusion. Articles that are entirely written in a language other than English, including their title, abstract, and main text, will be excluded because the research team do not have the resources to support translation.

### Search method

The following online databases will be systematically searched: CENTRAL, MEDLINE, PubMed, Embase, CINAHL, Scopus, and PsychINFO. In addition, the reference lists or bibliographies of the included full text articles will be searched in order to identify any articles that were not identified by searching the online databases. In accordance with the Preferred Reporting Items for Systematic review and Meta-Analysis Protocols (PRISMA-P) checklist ( Moher *et al.*, 2015), a draft of the search strings (i.e. keywords and Boolean operators) to be used for one electronic database (i.e. Embase) are provided in Table 1. The databases and search strings were selected, in consultation with an expert librarian, in order to source relevant research studies from a range of disciplines (e.g. emergency medicine, psychology).

**Table 1.** Search Strings.

	‘citizen rescue*’ OR ‘citizen responder*’ OR ‘community rescue*’ OR ‘community responder*’ OR ‘emergency responder*’ OR ‘first person on scene’ OR ‘first responder*’ OR ‘lay emergency medical technician*’ OR ‘lay rescue*’ OR ‘lay responder*’ OR ‘lay-person emergency medical technician*’ OR ‘lay-person rescue*’ OR ‘lay-person responder*’ OR ‘layperson emergency medical technician*’ OR ‘layperson rescue*’ OR ‘layperson responder*’ OR ‘voluntary emergency medical technician*’ OR ‘voluntary rescue*’ OR ‘voluntary responder*’ OR ‘volunteer emergency medical technician*’ OR ‘volunteer rescue*’ OR ‘volunteer responder*’
AND	‘pre-hospital medical emergenc*’ OR ‘prehospital medical emergenc*’ OR ‘prehospital emergenc*’ OR ‘pre-hospital emergenc*’ OR ‘out of hospital medical emergenc*’ OR ‘out of hospital emergenc*’ OR ‘out of hospital cardiac arrest*’

### Screening

All references will be imported into Endnote and duplicates removed. The first author (EH) will screen the title and abstracts of all the retrieved articles against the eligibility criteria. The second author (IO) will independently screen the title and abstracts of a sample of retrieved articles in accordance with the AMSTAR (A MeaSurement Tool to Assess systematic Reviews) 2 guidance ( Shea *et al.*, 2017). They will use Rayyan QCRI ( Ouzzani *et al.*, 2016). to support this process. The full text of every potentially relevant article will be obtained and examined for eligibility. The two authors will review the full text articles in line with the AMSTAR 2 guidance. Any disagreements about the exclusion of articles will be resolved through discussion. Where necessary, a third author (SM) will be engaged to make the final decision. In addition, the authors of the retrieved articles will be approached for additional information and clarification as needed. Reasons for the exclusion of articles will be noted. The search strategy and study selection process will be reported in accordance with the Preferred Reporting Items for Systematic reviews and Meta-Analyses (PRISMA) statement ( Liberati *et al.*, 2009; Moher *et al.*, 2009). A PRISMA flow diagram will be presented.

### Data extraction

One author (EH) will manually extract the data from the included studies and 10% of these will be randomly selected and checked for consistency by a second author (IO). Any discrepancies will be managed through discussion between the two authors or, where needed, consultation with a third author (SM). Information will be extracted via a data collection form for each of the SPIDER tool domains: Sample, Phenomena of Interest, Design, Evaluation, and Research type ( Cooke *et al.*, 2012). The form will also facilitate the extraction of general information about each study (e.g. title, date of publication, author details). The data collection form will be piloted and, where necessary, revised by the two authors responsible for data extraction ( Maidment *et al.*, 2018).

### Data synthesis

A qualitative synthesis will be performed, which entails developing new explanations and interpretations of the study findings ( Barnett-Page & Thomas, 2009). The specific qualitative synthesis methodology utilised will be a narrative synthesis. This approach entails developing textual descriptions that ‘tell the story’ of the qualitative and/or quantitative findings of the included studies ( Lucas *et al.*, 2007; Popay *et al.*, 2006). It comprises four main stages: (1) developing a preliminary synthesis of the results of the included studies, (2) exploring relationships within and between studies, (3) assessing the robustness of the synthesis, and (4) developing conclusions and recommendations ( Arai *et al.*, 2007; Lucas *et al.*, 2007; Popay *et al.*, 2006). Developing a preliminary synthesis can be achieved via a range of techniques, including producing a textual description of each study, organising studies into clusters, developing a table that describes each study, and creating a common rubric, such as transforming quantitative findings into a qualitative form ( Popay *et al.*, 2006). There are also several techniques for exploring relationships, including undertaking subgroup analyses, developing conceptual models, and creating visual representations, such as spider diagrams ( Popay *et al.*, 2006).

QSR International’s NVivo Software (Version 12) will be used to support the organisation and analysis of the data. One author (EH) will carry out the data analysis. In addition, peer assessment/second coding will be conducted in order to enhance the rigour and validity of the data analysis ( Heffernan *et al.*, 2018; Yardley, 2008). Specifically, a second author (TB) will independently analyse at least 10% of the papers included in the synthesis. The first and second author will meet to compare their analyses and to resolve any discrepancies through discussion. In addition, the first author will meet regularly with the research team during each stage of the synthesis, from the initial familiarisation with the data to the preparation of the written report, in order to obtain their perspectives and feedback, as well as to ensure that the analysis is not limited to the viewpoint or preconceptions of the first author.

### Quality appraisal

Study quality will be critically appraised using the Mixed Methods Appraisal Tool ( Hong *et al.*, 2018a). This validated tool was designed to enable systematic review authors to evaluate the quality of studies with diverse designs and paradigms ( Hong *et al.*, 2018b; Hong *et al.*, 2019; Souto *et al.*, 2015). It covers five methodological domains: qualitative research, randomised controlled trials, non-randomised studies, quantitative descriptive studies, and mixed methods studies. It is therefore suitable for this review, which is anticipated to primarily uncover qualitative and mixed methods studies. One author (EH) will assess the quality of papers selected for data extraction prior to inclusion in the review. A second author (TB) will assess 10% of the papers to check for consistency and to resolve any disagreements through discussion. A third author (SM) will be consulted as required. Furthermore, as part of the narrative synthesis process, the findings of the review itself will be critically appraised. This will involve examining the adequacy of the data supporting the findings and the contribution of lower quality studies to the findings. This appraisal will be carried out by one author and reviewed by a second.

### Dissemination

The results of the review will be reported at academic conferences, as well as in a peer reviewed journal using the PRISMA guidelines ( Liberati *et al.*, 2009). The findings will also be disseminated at a national event organised by the authors to inform key stakeholders (e.g. patients, CFRs, paramedics, general practitioners, and researchers) about the research. The findings will form part of the results of a larger study funded by an Applied Partnership Award from the Health Research Board of Ireland. The primary aim of the larger study is to develop recommendations regarding CFR data collection, integration, and analysis practices.

### Public and Patient Involvement

A panel of Patient and Public Involvement representatives, including CFRs, will contribute to this research. For example, during the study design phase, two representatives confirmed that the topic of this research is important to them because it can be difficult to recruit volunteers for CFR programs and to sustain their engagement in these programs over time. In addition, the representatives will be asked to provide their feedback and insights during the data analysis phase. Once the review has been completed, they will be involved in dissemination that targets CFRs and members of the public.

### Protocol registration

This protocol is registered with the International Prospective Register of Systematic Reviews (PROSPERO). Any amendments to the protocol (ID: CRD42019145316) will be recorded via PROSPERO.

### Study status

This review is ongoing. Preliminary searches have begun.

## Discussion

Volunteerism is a vital component of prehospital care for medical emergencies ( Whittaker *et al.*, 2015). Out-of-hospital cardiac arrest (OHCA) is the most time-critical medical emergency ( Ong *et al.*, 2018). Specifically, survival from OHCA is greatly reliant on the rapid provision of cardiopulmonary resuscitation (CPR) and defibrillation ( Hasselqvist-Ax *et al.*, 2015; Myat *et al.*, 2018). Therefore, OHCA management poses a considerable challenge for the EMS, particularly in remote or rural areas. Consequently, Voluntary First Response schemes have been established in many communities globally to improve emergency response times for OHCA patients ( Hollenberg *et al.*, 2013; Oving *et al.*, 2019). This intervention entails the mobilisation of volunteers, known as Community First Responders (CFRs), by the EMS or its equivalent to respond to OHCA events in their locality ( Barry *et al.*, 2019a). Since its inception, this intervention has grown in scope such that CFRs in many regions are now dispatched to a wide range of medical emergencies, including stroke, choking, and chest pain. Furthermore, the role of CFRs is becoming increasingly complex as, in addition to basic emergency care skills (e.g. CPR), they are often required to have specialised non-clinical skills, including resource management, communication, teamwork, and conflict resolution ( Phung *et al.*, 2018; Wilson *et al.*, 2015).

Developing an in-depth understanding of the motivation of individuals to participate in Voluntary First Response is vital to the optimisation of this intervention. The research to date indicates that a wide variety of factors can influence the motivation of individuals to join and remain engaged in Voluntary First Response schemes, including the availability of learning opportunities, feedback and recognition, psychological support, leadership, and consultation ( O’Meara *et al.*, 2012; Phung *et al.*, 2017; Timmons & Vernon-Evans, 2013). Therefore, the findings of this review could be used to inform in the design and organisation of these schemes, including their recruitment, retention, training, and psychological support processes. This, in turn, could have benefits for all stakeholders involved in Voluntary First Response, including the volunteers themselves, EMS personnel, and emergency physicians, as well as the patients, their family members, and their local communities.

## Data availability

### Underlying data

No data are associated with this paper.

### Reporting guidelines

Open Science Framework (OSF): PRISMA-P checklist for factors that motivate individuals to volunteer to be dispatched as first responders in the event of a medical emergency: A systematic review protocol, https://doi.org/10.17605/OSF.IO/SQVCZ ( Heffernan, 2019).

Data are available under the terms of the Creative Commons Zero “No rights reserved” data waiver (CC0 1.0 Public domain dedication).

## References

[ref-1] Arai L Britten N Popay J: Testing methodological developments in the conduct of narrative synthesis: a demonstration review of research on the implementation of smoke alarm interventions. *Evid Policy.* 2007;3(3):361–383. 10.1332/174426407781738029

[ref-2] Barnett-Page E Thomas J: Methods for the synthesis of qualitative research: a critical review. *BMC Med Res Methodol.* 2009;9(1):59. 10.1186/1471-2288-9-59 19671152 PMC3224695

[ref-3] Barry T Doheny MC Masterson S: Community first responders for out-of-hospital cardiac arrest in adults and children. *Cochrane Database Syst Rev.* 2019a;7:CD012764. 10.1002/14651858.CD012764.pub2 31323120 PMC6641654

[ref-4] Barry T González A Conroy N: Mapping the potential of community first responders to increase cardiac arrest survival. *Open Heart.* 2018;5(2):e000912 10.1136/openhrt-2018-000912 30402259 PMC6203054

[ref-5] Barry T Guerin S Bury G: Motivation, challenges and realities of volunteer community cardiac arrest response: a qualitative study of ‘lay’ community first responders. *BMJ Open.* 2019b;9(8):e029015. 10.1136/bmjopen-2019-029015 31399458 PMC6701604

[ref-6] Calcutt B: Valuing volunteers: Better understanding the primary motives for volunteering in Australian emergency services. Master of Philosophy Thesis, School of Management, Operations and Marketing, University of Wollongong.2019 Reference Source

[ref-7] Cooke A Smith D Booth A: Beyond PICO: the SPIDER tool for qualitative evidence synthesis. *Qual Health Res.* 2012;22(10):1435–1443. 10.1177/1049732312452938 22829486

[ref-8] Geri G Fahrenbruch C Meischke H: Effects of bystander CPR following out-of-hospital cardiac arrest on hospital costs and long-term survival. *Resuscitation.* 2017;115:129–134. 10.1016/j.resuscitation.2017.04.016 28427882

[ref-9] Hasselqvist-Ax I Riva G Herlitz J: Early cardiopulmonary resuscitation in out-of-hospital cardiac arrest. *N Engl J Med.* 2015;372(24):2307–2315. 10.1056/NEJMoa1405796 26061835

[ref-10] Heffernan E: Factors that motivate individuals to volunteer to be dispatched as first responders in the event of a medical emergency: A systematic review protocol.2019 10.17605/OSF.IO/SQVCZ PMC7236422 32490350

[ref-11] Heffernan E Coulson NS Ferguson MA: Development of the Social Participation Restrictions Questionnaire (SPaRQ) through consultation with adults with hearing loss, researchers, and clinicians: a content evaluation study. *Int J Aud.* 2018;57(10):791–799. 10.1080/14992027.2018.1483585 29966457

[ref-12] Hollenberg J Svensson L Rosenqvist M: Out-of-hospital cardiac arrest: 10 years of progress in research and treatment. *J Intern Med.* 2013;273(6):572–583. 10.1111/joim.12064 23480824

[ref-13] Hong QN Gonzalez-Reyes A Pluye P: Improving the usefulness of a tool for appraising the quality of qualitative, quantitative and mixed methods studies, the Mixed Methods Appraisal Tool (MMAT). *J Eval Clin Pract.* 2018b;24(3):459–467. 10.1111/jep.12884 29464873

[ref-14] Hong QN Pluye P Fàbregues S: Mixed methods appraisal tool (MMAT), version 2018.IC Canadian Intellectual Property Office, Industry Canada.2018a Reference Source

[ref-15] Hong QN Pluye P Fàbregues S: Improving the content validity of the mixed methods appraisal tool: a modified e-Delphi study. *J Cin Epidemiol.* 2019;111:49–59. 10.1016/j.jclinepi.2019.03.008 30905698

[ref-16] Kasper N Nabecker S Twerenbold GA: Keeping laypersons as first responders engaged: A qualitative, focus group interview study. *Resuscitation.* 2017;118:e10 10.1016/j.resuscitation.2017.08.037

[ref-17] Kindness P Fitzpatrick D Mellish C: An insight into the demands and stressors experienced by Community First Responders. *JPP.* 2014;6(7):362–369. 10.12968/jpar.2014.6.7.362

[ref-18] Liberati A Altman DG Tetzlaff J: The PRISMA statement for reporting systematic reviews and meta-analyses of studies that evaluate health care interventions: explanation and elaboration. *PLoS Med.* 2009;6(7):e1000100 10.1016/j.jclinepi.2009.06.006 19621070 PMC2707010

[ref-19] Lucas PJ Baird J Arai L: Worked examples of alternative methods for the synthesis of qualitative and quantitative research in systematic reviews. *BMC Med Res Methodol.* 2007;7(1):4. 10.1186/1471-2288-7-4 17224044 PMC1783856

[ref-20] Maidment DW Barker AB Xia J: A systematic review and meta-analysis assessing the effectiveness of alternative listening devices to conventional hearing aids in adults with hearing loss. *Int J Aud.* 2018;57(10):721–729. 10.1080/14992027.2018.1493546 30388942

[ref-21] Malta Hansen C Rosenkranz SM Folke F: Lay bystanders’ perspectives on what facilitates cardiopulmonary resuscitation and use of automated external defibrillators in real cardiac arrests. *J Am Heart Assoc.* 2017;6(3):e004572. 10.1161/JAHA.116.004572 28288975 PMC5524003

[ref-22] Masterson S Wright P O’Donnell C: Urban and rural differences in out-of-hospital cardiac arrest in Ireland. *Resuscitation.* 2015;91:42–47. 10.1016/j.resuscitation.2015.03.012 25818707

[ref-23] Mathiesen WT Bjørshol CA Braut GS: Reactions and coping strategies in lay rescuers who have provided CPR to out-of-hospital cardiac arrest victims: a qualitative study. *BMJ Open.* 2016;6(5):e010671. 10.1136/bmjopen-2015-010671 27225648 PMC4885284

[ref-24] Maurer H Masterson S Tjelmeland IB: When is a bystander not a bystander any more? A European survey. *Resuscitation.* 2019;136:78–84. 10.1016/j.resuscitation.2018.12.009 30572073

[ref-25] Moher D Liberati A Tetzlaff J: Preferred Reporting Items for Systematic Reviews and Meta-Analyses: The PRISMA Statement. *Ann Intern Med.* 2009;151(4):264–9, W64. 10.7326/0003-4819-151-4-200908180-00135 19622511

[ref-26] Moher D Shamseer L Clarke M: Preferred reporting items for systematic review and meta-analysis protocols (PRISMA-P) 2015 statement. *Syst Rev.* 2015;4(1):1. 10.1186/2046-4053-4-1 25554246 PMC4320440

[ref-27] Monsieurs KR Nolan JP Bossaert LL: European Resuscitation Council Guidelines for Resuscitation 2015: Section 1. Executive summary. *Resuscitation.* 2015;95:1–80. 10.1016/j.resuscitation.2015.07.038 26477410

[ref-28] Myat A Song KJ Rea T: Out-of-hospital cardiac arrest: current concepts. *Lancet.* 2018;391(10124):970–979. 10.1016/S0140-6736(18)30472-0 29536861

[ref-29] O’Meara P Tourle V Rae J: Factors influencing the successful integration of ambulance volunteers and first responders into ambulance services. *Health Soc Care Community.* 2012;20(5):488–496. 10.1111/j.1365-2524.2011.01055.x 22313138

[ref-30] Ong ME Perkins GD Cariou A: Out-of-hospital cardiac arrest: prehospital management. *Lancet.* 2018;391(10124):980–988. 10.1016/S0140-6736(18)30316-7 29536862

[ref-31] Orkin AM VanderBurgh D Ritchie SD: Community-Based Emergency Care: A Model for Prehospital Care in Remote Canadian Communities. *CJEM.* 2016;18(5):385–388. 10.1017/cem.2016.339 27452641

[ref-32] Ouzzani M Hammady H Fedorowicz Z: Rayyan-a web and mobile app for systematic reviews. *Syst Rev.* 2016;5(1):210. 10.1186/s13643-016-0384-4 27919275 PMC5139140

[ref-33] Oving I Masterson S Tjelmeland I: Inventory of first-response treatments after out-of-hospital cardiac arrest in Europe. *Resuscitation.* 2019;142(1):e2–e3. 10.1016/j.resuscitation.2019.06.017 PMC6916130 31842928

[ref-34] Phung VH Trueman I Togher F: Community first responders and responder schemes in the United Kingdom: systematic scoping review. *Scand J Trauma Resusc Emerg Med.* 2017;25(1):58. 10.1186/s13049-017-0403-z 28629382 PMC5477292

[ref-35] Phung VH Trueman I Togher F: Perceptions and experiences of community first responders on their role and relationships: qualitative interview study. *Scand J Trauma Resusc Emerg Med.* 2018;26(1):13. 10.1186/s13049-018-0482-5 29402312 PMC5800091

[ref-36] Popay J Roberts H Sowden A: Guidance on the conduct of narrative synthesis in systematic reviews. A Product from the ESRC Methods Programme. Version 1,2006;b92 10.13140/2.1.1018.4643

[ref-37] Rice S Fallon B: Retention of volunteers in the emergency services: Exploring interpersonal and group cohesion factors. *AJEM.* 2011;26(1):18 Reference Source

[ref-38] Roberts A Nimegeer A Farmer J: The experience of community first responders in co-producing rural health care: in the liminal gap between citizen and professional. *BMC Health Serv Res.* 2014;14(1):460. 10.1186/1472-6963-14-460 25326796 PMC4283089

[ref-39] Rørtveit S Meland E: First responder resuscitation teams in a rural Norwegian community: sustainability and self-reports of meaningfulness, stress and mastering. *Scand J Trauma Resusc Emerg Med.* 2010;18(1):25. 10.1186/1757-7241-18-25 20441592 PMC2879234

[ref-40] Savastano S Vanni V: Cardiopulmonary resuscitation in real life: the most frequent fears of lay rescuers. *Resuscitation.* 2011;82(5):568–571. 10.1016/j.resuscitation.2010.12.010 21333434

[ref-70] Shea BJ Reeves BC Wells G: AMSTAR 2: a critical appraisal tool for systematic reviews that include randomised or non-randomised studies of healthcare interventions, or both. *BMJ.* 2017;358::j4008. 10.1136/bmj.j4008 28935701 PMC5833365

[ref-41] Snyder M Omoto AM: Volunteerism: Social issues perspectives and social policy implications. *Soc Issues Policy Rev.* 2008;2(1):1–36. 10.1111/j.1751-2409.2008.00009.x

[ref-42] Souto RQ Khanassov V Hong QN: Systematic mixed studies reviews: updating results on the reliability and efficiency of the Mixed Methods Appraisal Tool. *Int J Nurs Stud.* 2015;52(1):500–501. 10.1016/j.ijnurstu.2014.08.010 25241931

[ref-50] Stirling C Bull R: Collective agency for service volunteers: a critical realist study of identity representation. *Administration & Society.* 2011;43(2):193–215. 10.1177/0095399711400046

[ref-43] Timmons S Vernon-Evans A: Why do people volunteer for community first responder groups? *Emerg Med J.* 2013;30(3):e13. 10.1136/emermed-2011-200990 22508195

[ref-44] Toyokuni Y Suzukawa M Yamashita K: Introduction of the community first responder system into Japan: is that possible? *Int J Emerg Med.* 2013;6(1):34. 10.1186/1865-1380-6-34 24079305 PMC3852586

[ref-45] Truong HT Low LS Kern KB: Current Approaches to Cardiopulmonary Resuscitation. *Curr Probl Cardiol.* 2015;40(7):275–313. 10.1016/j.cpcardiol.2015.01.007 26071014

[ref-46] Whittaker J McLennan B Handmer J: A review of informal volunteerism in emergencies and disasters: Definition, opportunities and challenges. *Int J Disaster Risk Reduct.* 2015;13:358–368. 10.1016/j.ijdrr.2015.07.010

[ref-47] Wilson MH Habig K Wright C: Pre-hospital emergency medicine. *Lancet.* 2015;386(10012):2526–2534. 10.1016/S0140-6736(15)00985-X 26738719

[ref-48] Yardley L: Demonstrating Validity in Qualitative Psychology. In J. A. Smith (Ed.), *Qualitative Psychology: A Practical Guide to Research Methods (Vol. 2, pp. 235-251)* London: Sage Publications.2008 Reference Source

[ref-49] Zijlstra JA Beesems SG De Haan RJ: Psychological impact on dispatched local lay rescuers performing bystander cardiopulmonary resuscitation. *Resuscitation.* 2015;92:115–121. 10.1016/j.resuscitation.2015.04.028 25957944

