# Supplementary material for: Factors that motivate individuals to volunteer to be dispatched as first responders in the event of a medical emergency: A systematic review protocol
Source: HRB Open Res. 2020 Sep 14;2:34. Originally published 2019 Dec 3. [Version 2] doi: 10.12688/hrbopenres.12969.2 (PMC7236422; doi:10.12688/hrbopenres.12969.2)
Supplement: Peer review history

# Peer review: Reviewer response for version 1

**DOI:** 10.21956/hrbopenres.14054.r27393

**Published:** 2020-05-18

**Authors:** Katie Dainty

**Affiliations:** 1 Institute of Health Policy Management and Evaluation, Dalla Lana School of Public Health, University of Toronto, Toronto, Canada; 2 North York General Hospital, Toronto, Canada

Thank you for the opportunity to review this SR protocol. This is an important topic and the authors have done an excellent job of outlining a thorough protocol. I just have a couple of comments/areas for clarification:

- I would add Scotland to your list of countries with CFR programs; the Sandpiper Wildcat program is very robust in the Grampian region there.
- You mention that you will consider qualitative, mixed method and any other study type that answers the research question but you only outline a qualitative analysis approach. It would be good to clarify how you will handle other types of data that may appear (survey for example).
- Why will only one author work on the analysis before bringing it to the team? I think it would be more rigorous to have this initial synthesis discussions ​​​​​with multiple team members (similar to double coding the first 3-4 interviews in a qualitative analysis).
- Lastly you mention in your dissemination section that Patient and Public Involvement representatives will involved in disseminating the findings but I would STRONGLY encourage you to involve them throughout the study in a more integrated knowledge translation approach. Their insight will be invaluable on this topic.

Best of luck with this SR - I look forward to seeing the results!

Is the study design appropriate for the research question?

Yes

Is the rationale for, and objectives of, the study clearly described?

Yes

Are sufficient details of the methods provided to allow replication by others?

Yes

Are the datasets clearly presented in a useable and accessible format?

Not applicable

Reviewer Expertise:

Community response to out-of-hospital cardiac arrest; specifically bystander CPR; qualitative social science; patient-centred outcomes in health care.

I confirm that I have read this submission and believe that I have an appropriate level of expertise to confirm that it is of an acceptable scientific standard.

# Peer review: Reviewer response for version 1

**DOI:** 10.21956/hrbopenres.14054.r27282

**Published:** 2020-04-20

**Authors:** Christopher M. Smith

**Affiliations:** 1 University of Warwick, Coventry, UK

A detailed and well-thought-out protocol. I have two minor comments:

1. Those joining statutory/regulatory CFR schemes might (or might not) have very different reasons for volunteering than those joining up to ad-hoc schemes (e.g. GoodSAM, Pulsepoint, Heartrunner). A little more clarity regarding whether you plan to study these types of schemes separately or put all the results together.
2. Search strategy: how do you plan to identify articles that your original electronic searches fail to identify. e.g. bibliography/reference reviews of included full-texts; related articles features in PubMed or Google Scholar etc.

Is the study design appropriate for the research question?

Yes

Is the rationale for, and objectives of, the study clearly described?

Yes

Are sufficient details of the methods provided to allow replication by others?

Partly

Are the datasets clearly presented in a useable and accessible format?

Yes

Reviewer Expertise:

Out-of-hospital cardiac arrest.

I confirm that I have read this submission and believe that I have an appropriate level of expertise to confirm that it is of an acceptable scientific standard.
